# CHILDREN’S HEALTH: Soy Formula of “Minimal Concern”

**DOI:** 10.1289/ehp.118-a335

**Published:** 2010-08

**Authors:** Tanya Tillett

**Affiliations:** **Tanya Tillett**, MA, of Durham, NC, is a staff writer/ editor for *EHP*. She has been on the *EHP* staff since 2000 and has represented the journal at national and international conferences

In May 2010 the National Toxicology Program (NTP) released its draft opinion on the potential of soy infant formula to cause adverse human developmental effects, labeling its concern level as “minimal,” or a 2 on the 5-level scale used by the NTP. This draft opinion was based primarily on the conclusions of an expert panel evaluation of the existing literature in humans and laboratory animals, although many of the studies included in the review were not considered by the expert panel to be useful for the evaluation. For instance, none of the 80 human studies reviewed were considered “high utility,” and only 28 were considered of “limited utility.”^1^

Soy formula is a relatively small component of the U.S. formula market, comprising 12% of sales between June and September 2009.^1^ Infants fed soy formula receive higher daily intakes of isoflavones (plant-derived compounds with biological activity similar to that of estrogen) than not just other infants but also subpopulations (such as Asians and vegans) that consume soy-rich diets.^1^

Results from some animal studies (e.g., Cimafranca et al.^2^) point to impairment of reproductive development in female rodents treated with genistein, the best studied soy isoflavone. However, according to the panel, very few studies have analyzed the potential reproductive or other long-term health effects in people who consumed soy formula during infancy. This lack of data made it impossible for the expert panel to assess whether soy formula causes adverse effects in humans. At the same time, the evidence of effects in animals made it impossible to find soy formula free of any health threat.

Marisa Salcines, manager of communications for the International Formula Council, says the organization agrees with the “minimal” concern level rating. “Soy formulas have been used for over fifty years without reports of negative reproductive or developmental effects,” she says. Ed Carney, a developmental toxicologist at the Dow Chemical Company and member of the NTP Board of Scientific Counselors (which reviewed the draft report in May), agrees. “Decades of real-life clinical experience have not resulted in any overt ‘red flags’ for developmental toxicity,” he says. “Given the high levels of exposures and vast numbers of children exposed for so many years, one would expect that some hints of adverse effects [would have been seen] if they were really there.” Some research even suggests beneficial protection against cancer in rodents fed soy protein isolate.^3^

Despite this track record, Elaine Faustman, a professor of environmental and occupational health sciences at the University of Washington School of Public Health and member of the NTP Board of Scientific Counselors, says more weight should be attributed to data showing estrogenic effects in animal studies. “The data should not be downplayed or discounted,” she says, cautioning that in addition to soy formula, children may be exposed to soy in other foods as well. “We should consider the variety of . . . mixed, real-world exposures such as cereal, yogurt, soy milk, and other foods in [the older] infant diet,” she explains.

The draft brief outlines proposed future research, which will focus on exposing animals to a mixture of isoflavones to better mirror infants’ actual exposure to soy formula. Carney agrees with this approach, saying it should include feeding complete soy formula to animals, but questions the relevance of the rodent models used in the majority of the animal studies reviewed. He says animal studies should include pigs, which “are much better models of humans exposed to soy formula.”

Two ongoing studies in human infants may fill some of the data gaps, although they will not necessarily address the potential long-term impacts on female reproductive function identified in the laboratory animal studies. The ongoing Arkansas Children’s Nutrition Center Prospective Cohort Study (The Beginnings Study) is following children from age 4–8 weeks through 6 years,^4^ while the recently launched Infant Feeding and Early Development (IFED) study, conducted by NIEHS researchers in collaboration with pediatricians at the Children’s Hospital of Philadelphia, will follow 600 infants over their first two years of life.

“The IFED study uses detailed, specific measures of estrogen exposure, similar to those used in the laboratory, to evaluate human infants,” says principal investigator Walter Rogan, head of the NIEHS Pediatric Epidemiology Group. “Their main exposure to estrogen has been from their own mothers, who had very high levels of estrogen in their blood while they were pregnant.” Rogan says normal infants respond to this estrogen—for example, all newborns have breast buds—but the effects wane after birth. “We think that a slower disappearance of those effects is a very sensitive way of measuring whether the baby is exposed to any estrogen.” Rogan says the IFED study will aid in translating the effects seen in laboratory experiments into predictions for human health, not just for soy formula but also for other chemicals such as phthalates and bisphenol A.

Still more studies will be necessary, says Susan Schantz, chair of the Pharmacology/Toxicology Division at the University of Illinois at Urbana–Champaign. “Adverse effects of early exposure to the dietary estrogens in soy may not manifest themselves during the first year of life,” she explains. “In order to completely and adequately assess the health risks, infants who consume soy infant formula need to be followed prospectively to puberty and beyond.”

So what is the bottom line on soy formula use? The American Academy of Pediatrics states there is no conclusive evidence that dietary soy isoflavones harm human development, reproduction, or endocrine function, but notes that soy formula should be used only in limited circumstances in place of cow’s milk formula, such as in cases of infant lactase deficiency.^5^ Meanwhile, the final NTP opinion is expected by fall 2010.

## Figures and Tables

**Figure:**
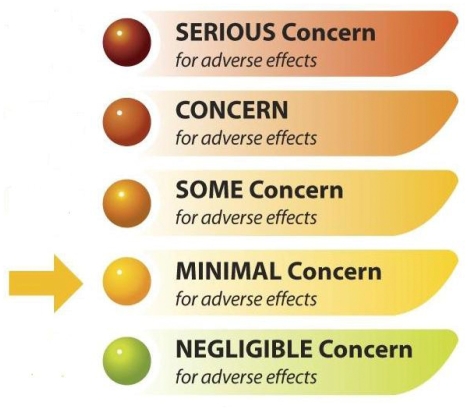
The NTP, which assigns levels of concern on a 5-point scale, has judged soy formula to be of “minimal concern” for human health.
